# A flow cytometry-based assay for serological detection of anti-spike antibodies in COVID-19 patients

**DOI:** 10.1016/j.xpro.2021.100671

**Published:** 2021-06-19

**Authors:** Yun Shan Goh, Lisa F.P. Ng, Laurent Renia

**Affiliations:** 1 Infectious Diseases Laboratories (ID Labs), Agency for Science, Technology and Research (A∗STAR), Immunos, Biopolis, Singapore 138648, Singapore; 2 Department of Biochemistry, Yong Loo Lin School of Medicine, National University of Singapore, Singapore 117596, Singapore; 3 National Institute of Health Research, Health Protection Research Unit in Emerging and Zoonotic Infections, University of Liverpool, Liverpool, United Kingdom; 4 Institute of Infection, Veterinary and Ecological Sciences, University of Liverpool, Liverpool, United Kingdom; 5 Lee Kong Chian School of Medicine, Nanyang Technological University, 11 Mandalay 20 Road, Singapore 308232, Singapore

**Keywords:** Cell Biology, Flow Cytometry/Mass Cytometry, Health Sciences, Immunology, Molecular Biology, Antibody

## Abstract

One of the key public health strategies in coronavirus 2019 disease (COVID-19) management is the early detection of infected individuals to limit the transmission. As a result, serological assays have been developed to complement PCR-based assays. Here, we report the development of a flow cytometry-based assay to detect antibodies against full-length SARS-CoV-2 spike protein (S protein) in patients with COVID-19. The assay is time-efficient and sensitive, being able to capture the wider repertoire of antibodies against the S protein.

For complete details on the use and execution of this protocol, please refer to Goh et al. (2021).

## Highlights

• Cloning strategy allows insertion of SARS-CoV-2 spike gene into pHIV-eGFP
• Transfection of transfer and packaging plasmids enables production of viral particles
• Cells are transduced to stably express SARS-CoV-2 spike protein on the cell surface
• Flow cytometry assay detects specific antibodies against SARS-CoV-2 spike protein

## Graphical abstract

**Figure:**
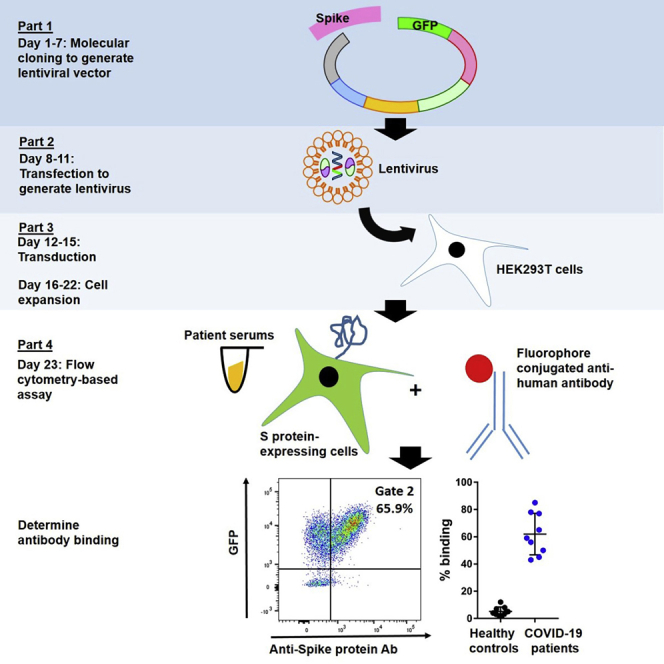

## Before you begin

The protocol consists of four main parts. Three of the main parts are preparation steps to generate the S protein-expressing cells for the assay itself: (1) generation of transfer plasmid for transfection, (2) transfection to generate lentiviral particles, (3) transduction to generate S protein-expressing cells. The final part (4) involves the flow cytometry-based assay to detect specific antibodies against S protein.

### Generation of transfer plasmid for transfection to generate lentiviral particles

**Timing: 1 week**

***Note:*** The DNA sequence, encoding for the full length S protein, is codon-optimized (Table 1) and is chemically synthesized by Genscript. The lead time for the chemical synthesis of the DNA sequence by Genscript is about 2–3 weeks.

**Table 1:** DNA sequence of codon-optimized SARS-CoV-2 S gene and primers used to sequence full length SARS-CoV-2-S protein

SARS-CoV-2 S gene Codon-optimized	ATGTTTGTATTCTTGGTACTTCTCCCATTGGTATCTTCTCAATGCGTTAACC TTACCACACGCACCCAACTGCCCCCGGCCTACACTAATAGCTTTACGCG GGGTGTCTACTATCCCGACAAAGTCTTTCGATCCAGTGTGCTCCACTCCA CCCAGGATCTTTTCCTTCCCTTTTTTTCTAATGTTACGTGGTTCCACGCAAT CCATGTATCCGGTACGAATGGGACAAAACGCTTTGACAATCCAGTGCTG CCATTTAATGATGGAGTGTACTTTGCATCTACCGAGAAGAGTAACATCATCA GAGGATGGATCTTCGGAACGACCTTGGACTCCAAAACGCAATCCTTGCTT ATCGTTAACAATGCAACGAATGTTGTCATCAAAGTTTGCGAATTCCAATTCT GTAACGATCCCTTCCTCGGTGTTTATTATCATAAAAATAATAAATCTTGGAT GGAAAGTGAGTTCCGCGTATACAGTTCCGCCAATAATTGTACCTTCGAA TACGTAAGTCAACCGTTCTTGATGGATCTGGAAGGTAAACAGGGTAACT TTAAGAACCTTCGGGAGTTTGTTTTTAAGAACATAGACGGCTACTTTAAGA TCTATAGTAAACATACGCCAATTAACTTGGTTAGAGATCTCCCGCAGGGG TTTTCAGCATTGGAGCCGCTCGTCGACCTCCCCATAGGTATAAATATAAC TCGGTTTCAAACACTGCTGGCGCTCCACCGCAGCTACCTGACGCCTGG GGATTCTTCTTCCGGTTGGACTGCAGGCGCTGCTGCATATTATGTAGGG TACCTGCAACCGAGAACCTTTCTCCTTAAGTACAACGAGAATGGCACTA TTACGGACGCTGTCGATTGTGCACTCGACCCCTTGAGTGAGACGAAGT GTACACTGAAAAGCTTTACTGTTGAAAAGGGAATATATCAGACATCCA ACTTTAGAGTTCAGCCAACAGAATCCATCGTTCGATTTCCCAATATTAC AAATCTCTGTCCGTTCGGAGAGGTCTTTAATGCTACCCGATTCGCGTC AGTATACGCCTGGAACAGAAAGAGAATTTCTAACTGTGTTGCAGATTA TAGTGTCCTGTATAATTCTGCGTCTTTTAGCACTTTTAAGTGCTACGGC GTTAGCCCCACTAAGTTGAACGACCTTTGTTTCACTAACGTGTATGCCG ACTCATTCGTCATAAGAGGCGACGAAGTTAGACAAATTGCACCGGGCC AGACGGGAAAGATTGCGGACTACAACTATAAATTGCCTGACGACTTT ACAGGATGTGTCATCGCCTGGAATAGTAATAACCTTGACTCCAAAGT CGGTGGCAATTACAATTACTTGTACCGGCTGTTCAGGAAGTCTAATC TCAAACCTTTTGAGCGAGATATCAGCACGGAAATTTATCAAGCTGGT AGCACTCCATGTAACGGGGTTGAGGGTTTTAATTGTTATTTTCCATT GCAATCATATGGATTCCAACCGACTAACGGTGTTGGGTATCAACCA TACAGAGTGGTGGTTTTGTCATTTGAACTTCTGCATGCCCCTGCAAC AGTGTGCGGACCGAAGAAGAGTACGAACCTTGTAAAGAACAAGTG CGTCAACTTCAACTTTAATGGTCTGACGGGTACCGGCGTTCTGAC GGAATCCAATAAAAAGTTCTTGCCCTTTCAGCAGTTCGGGCGAGA TATCGCCGACACTACTGATGCGGTGCGAGATCCTCAGACACTTGA GATCCTCGATATTACCCCATGTAGTTTTGGTGGTGTGTCTGTGATT ACACCCGGCACCAATACGTCAAATCAGGTCGCAGTCTTGTACCAA GACGTGAACTGCACCGAAGTTCCTGTAGCCATTCACGCTGATCAA TTGACACCGACATGGAGGGTGTACTCCACCGGATCTAACGTGTT CCAGACCCGCGCGGGGTGTCTTATCGGCGCAGAACATGTGAACA ACTCTTACGAATGTGATATTCCTATCGGTGCAGGCATCTGTGCCT CATACCAGACACAAACGAACTCACCAAGGAGGGCAAGGTCAGT AGCCTCACAAAGCATAATAGCCTATACGATGAGTCTTGGTGCG GAGAACTCTGTGGCGTACTCTAATAACTCTATCGCCATACCGAC TAACTTCACCATTTCTGTTACGACCGAGATCCTCCCAGTTTCCATG ACTAAGACAAGTGTGGATTGTACAATGTACATCTGCGGCGACA GTACTGAGTGCAGTAACCTGCTTCTGCAGTACGGGTCCTTCTGCA CACAACTTAACCGGGCGCTGACTGGTATAGCGGTTGAACAAGACA AGAACACTCAAGAGGTCTTCGCACAAGTAAAACAAATATACAAAAC ACCACCTATTAAAGATTTCGGCGGGTTTAATTTTAGCCAAATCCTTC CAGACCCCAGCAAACCCTCTAAGCGCAGCTTCATTGAGGATCTGC TGTTTAACAAGGTCACCCTGGCAGACGCGGGCTTTATCAAGCAA TACGGTGACTGCCTGGGGGATATCGCGGCTCGAGACCTTATATG TGCGCAAAAATTTAATGGACTTACCGTACTTCCTCCATTGCTGA CTGACGAGATGATAGCACAGTATACATCTGCACTGCTCGCCGGT ACAATTACATCAGGGTGGACATTTGGGGCGGGAGCTGCGCTCC AGATACCGTTCGCGATGCAGATGGCGTATAGGTTTAATGGAA TTGGTGTCACGCAAAACGTTCTCTATGAAAACCAGAAGCTGATA GCAAATCAGTTCAATTCCGCGATTGGTAAGATACAAGATTCATT GTCTAGTACGGCCTCTGCACTCGGAAAACTCCAAGATGTAGTG AACCAAAACGCCCAAGCCCTGAATACACTCGTAAAACAGCTC TCTAGTAATTTTGGGGCCATTTCCTCCGTATTGAACGACATCTT GAGTCGCTTGGATAAGGTAGAAGCAGAAGTACAAATTGACCG GTTGATCACGGGCAGACTTCAATCACTTCAGACTTATGTTACT CAGCAGCTTATACGAGCTGCAGAAATTCGCGCCTCTGCGAA CCTGGCCGCCACTAAAATGTCAGAATGTGTACTGGGACAGAG CAAACGGGTGGATTTCTGCGGAAAGGGCTATCATCTGATGAG TTTTCCCCAGTCTGCGCCTCATGGTGTAGTATTTCTTCATGTCAC ATATGTACCAGCCCAAGAAAAAAATTTCACAACGGCGCCC GCGATTTGCCATGACGGTAAGGCGCATTTTCCTCGCGAGGGCGTT TTCGTGTCTAACGGTACTCACTGGTTCGTAACACAGCGAAACTTTTA CGAGCCTCAGATAATCACGACGGATAACACATTTGTCTCCGGCA ACTGCGATGTGGTCATCGGTATAGTGAACAATACGGTATATGATC CGCTGCAGCCAGAGCTCGACAGTTTCAAGGAGGAGCTTGACAAA TACTTTAAGAACCATACCTCCCCAGACGTAGACCTCGGAGACAT ATCTGGTATCAATGCCTCCGTGGTTAACATACAAAAGGAGATAG ATAGACTGAATGAGGTGGCGAAGAATCTGAATGAGTCTCTCAT AGATCTGCAGGAACTCGGTAAATATGAACAATACATCAAGTGG CCTTGGTACATCTGGCTGGGGTTCATAGCGGGCCTGATCGCG ATCGTGATGGTAACTATAATGTTGTGTTGCATGACCTCCTGCTG CTCATGCCTTAAAGGTTGTTGTTCTTGCGGGAGCTGCTGCAAGT TCGATGAGGATGATTCAGAACCCGTCTTGAAG GGCGTAAAACTTCACTATACGTAA	
Primers used to sequence full length SARS-CoV-2-S protein	EF1aFor	GGATCTTGGTTCATTCTCAAG
	SPseqF1	GTACCTGCAACCGAGAAC
	SPseqF2	GGCGTTCTGACGGAATC
	SPseqF3	GCAATACGGTGACTGCC
	SPseqF4	CGTGTCTAACGGTACTCAC
	SPseqR1	GTTCTCGGTTGCAGGTAC
	IRESrev	CATATAGACAAACGCACACC

Below details the protocol to clone the S gene into the transfer plasmid, pHIV-eGFP.

For more info on the manufacturer’s instructions, please refer to Table 2 at the end of this section.

**Table:** 

Overview		
Day 1	Step 1	• Preparation of vector
Day 2	Step 2	• Preparation of insert
Day 3	Step 3 and 4	• Ligation of insert fragment into vector backbone • Transformation of ligation mix into chemically competent bacterial cells
Day 4	Step 5	• Colony PCR
Day 5	Step 6	• Plasmid extraction

**Figure 1:**
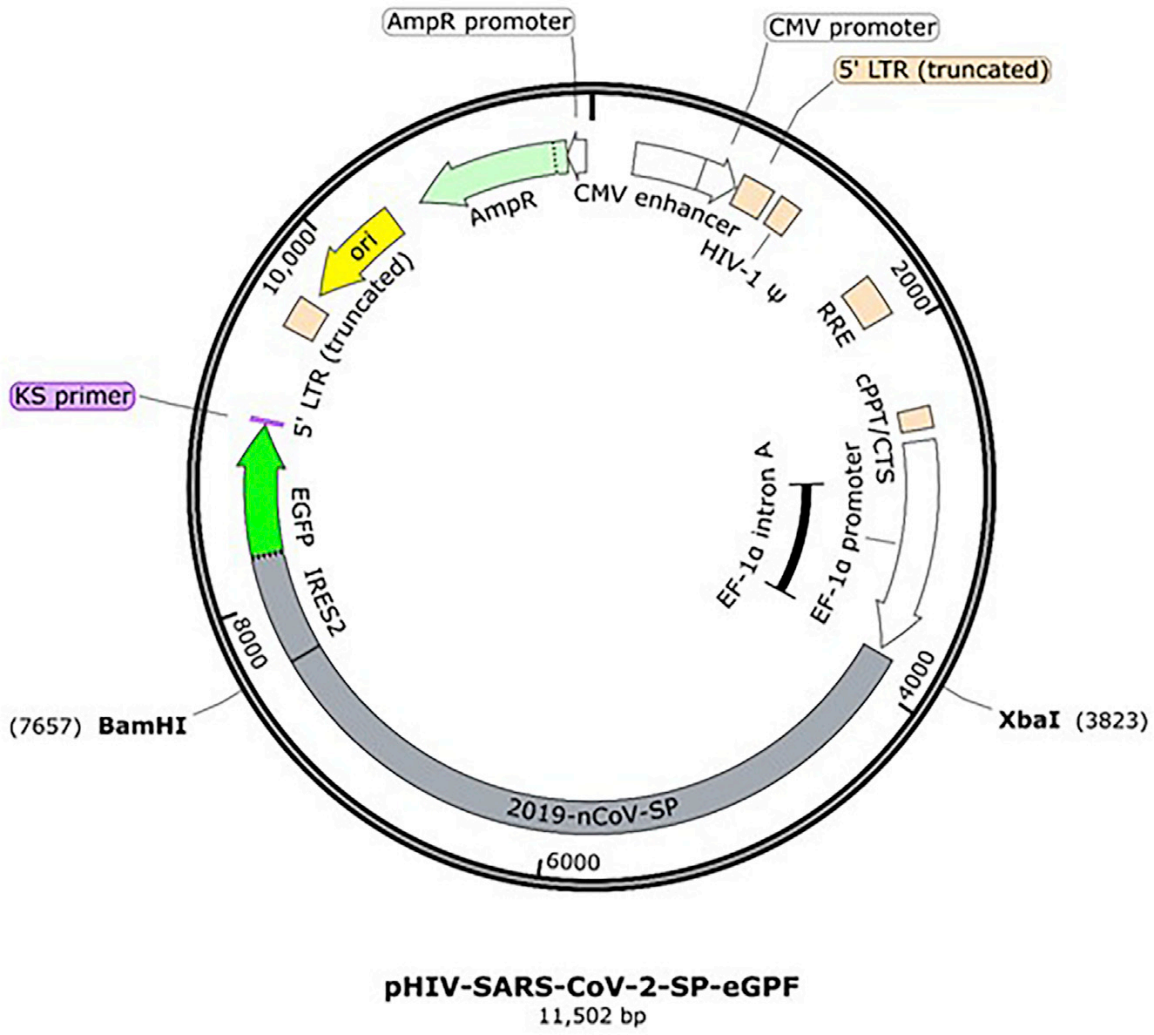
Plasmid map of pHIV-eGFP S gene is inserted between the XbaI and BamHI sites.

***Note:*** We advise to first calculate the amount of ligation reactions intended for Step 3a. If the amount of gel-extracted DNA falls below the calculated amount, repeat the enzymatic digest and gel-extraction.

1. Day 1: Preparation of the Vector backbone, pHIV-eGFP (Figure 1)
  a. Double-digest the vector with XbaI and BamHI for 2 h at 37°C as below:

**Table:** 

Reagent	Amount
Vector, pHIV-eGFP	5 μg
NEB Cutsmart buffer (10×)	2 μL
XbaI (20 U/μL)	0.5 μL
BamHI (20 U/μL)	0.5 μL
Nuclease-free water	Complete to 20 μL

    ***Note:*** We have used 5 μg vector for digestion to ensure there is sufficient cleaved fragment to proceed to the next step. A lower vector DNA (such as 1–2 μg) can be used too.
    ***Note:*** The amount of enzymes can be increased to a maximum of 10% of the total reaction volume. More than 10% might affect the digestion, due to the glycerol content.
    ***Alternatives:*** XbaI and BamHI enzymes from other suppliers, such as Promega, (#R6181 and #R6021 respectively) can be used.
  b. Run the digest on 0.8% agarose TAE gel at 100 V for 90 min.
    ***Note:*** Run 1 kb DNA marker.
    ***Note:*** Run non-digested vector as a control. If the digest is not complete, the band profile will be similar to the control, with more bands in addition to the fragments of interest. In this case, set up the reaction with 0.5 μL more of each enzyme, or increase the enzyme volume to a maximum of 10% of the total reaction volume.
    ***Note:*** The digest can be divided and run in 2–3 wells to allow better resolution on the gel.
  c. Gel-extract the vector backbone (∼7.6 kb), using the NEB’s Monarch gel extraction kit.
    ***Alternatives:*** Other gel extraction kits can be used, such as QIAquick Gel Extraction Kit (QIAGEN #28704).
  d. Quantify the DNA using a spectrophotometer.
  e. Store at −20°C until use.

2. Day 2: Preparation of the insert (encoding the S protein)
  a. Double-digest the insert with XbaI and BamHI for 2 h at 37°C, as described in step 1a.
    ***Note:*** The chemically synthesized insert (by Genscript) is designed to be flanked by XbaI at the 5′ end and BamHI at the 3′ end.
  b. Run the digest on 0.8% agarose TAE gel at 100 V for 90 min.
  c. Gel-extract the insert fragment (∼3.8 kb), as described in step 1c.
  d. Quantify the DNA using a spectrophotometer.
  e. Store at −20°C until use.

3. Day 3: Ligation of insert fragment into vector backbone
  a. Set up the ligation reaction as below:

**Table:** 

Reagent	Amount
XbaI/BamHI-digested Vector, pHIV-eGFP	100 ng
XbaI/BamHI-digested insert	at 3:1 molar excess over the vector
Rapid Ligation buffer (5X)	4 μL
T4 ligase (5 U/μL)	1 μL
Nuclease-free water	Complete to 20 μL
  b. Incubate for 5–20 min at 20°C–22°C.

***Note:*** The ligation can also be incubated at 16°C for 12–16 h.

***Note:*** In parallel, set a ligation negative control reaction, where only the digested vector is included and no insert is included. The double-digested vector has incompatible ends, hence ligation should not be possible.

4. Transformation of ligation mix into chemically competent bacterial cells.
  a. Add 2.5 μL ligation mix to 25 μL XL10-gold competent cells.
  b. Transform according to the manufacturer’s instructions.
  c. Plate the mixture on LB-ampicillin agar plates (100 μg/mL ampicillin).
  d. Incubate the LB-ampicillin agar plates at 37°C for 12–16 h.

***Note:*** Alternatively, other competent cells with low recombination capacity can be used such as top10 (Thermo Fisher Scientific #C404010).

***Note:*** The plasmid, pHIV-eGFP, contains an ampicillin resistance cassette.

***Note:*** There should be no colonies on the plate transformed with the ligation negative control reaction, where only the vector is included. Colonies on this plate would mean either there is inefficient digestion or inefficient gel extraction (possibly because the digested fragments have not been resolved well on the gel. In this case, repeat step 1).

5. Day 4: perform a colony PCR to screen bacterial colonies containing the plasmid with insert, using SPseqF4 and IRESrev primers (Table 1) and Phusion DNA polymerase.
  a. Set up PCR mix as below:

**Table:** 

Reagents	Amount
Primer, SPseqF4 (10 μM; final concentration 200 nM)	0.5 μL
Primer, IRESrev (10 μM; final concentration 200 nM)	0.5 μL
dNTP (5 mM, final concentration 100 μM)	0.5 μL
HF Buffer (5X)	5 μL
MgCl_2_ solution (50 nM; final concentration 2 nM)	1 μL
Phusion DNA polymerase (2 U/μL)	0.25 μL
Nuclease-free water	Complete to 25 μL

    ***Alternatives:*** Other polymerases, such AmpliTaq polymerase (Thermo Fisher Scientific Cat# N8080153) can also be used.
  b. Use a micropipette tip to touch the colony, dab onto a LB-ampicillin agar plate (100 μg/mL ampicillin, Sigma-Aldrich Cat# A0166) and then mix in the PCR reaction mix for each tube.
    ***Note:*** Ensure that the picked colonies on the LB-ampicillin agar plate are numbered.
  c. Perform the PCR with the below cycling conditions:

**Table:** 

Step	Cycle	Temperature	Time
Initial denaturation	1	98°C	2 min
Denaturation	25–30	98°C	30 s
Annealing ∗		55°C	30 s
Extension		72°C	30 s
Final extension	1	72°C	2 min

∗Annealing temperature indicated is optimized based on the Tm of the SPseqF4 and IRESrev primers. Typically, annealing temperature is Tm – 5°C.
  d. Analyze the colony PCR by running a 1% agarose TAE gel. A band of approximately 600 bp should be present if the insert is successfully cloned into the vector.
  e. Pick 3–5 positive colonies, each colony into 3–5 mL LB-ampicillin broth. Incubate on shaking (250 rpm) at 37°C for 12–16 h.

***Note:*** Incubation should be no longer than 16 h as the colonies might be overgrown, affecting the DNA recovery.

6. Day 5: Plasmid extraction
  a. Extract plasmids, using QIAprep Spin Miniprep kit.
    ***Alternatives:*** Other plasmid extraction kits, such NucleoSpin Plasmid Mini kit (Macherey Nagel Cat# 740588.50) can also be used.
    ***Note:*** The extraction of the plasmid can be scaled up by extracting from a 100 mL culture, using a QIAGEN plasmid Maxi kit (#12162).
  b. Sequence extracted plasmid using primers in Table 1.

**Table 2:** Links to manufacturer’s instructions

Step	Links to manufacturer’s instructions
1a	• https://www.neb.sg/products/r0145-xbai#Protocols,%20Manuals%20&%20Usage • https://www.neb.sg/products/r3136-bamhi-hf#Protocols,%20Manuals%20&%20Usage • https://www.neb.sg/protocols/2012/12/07/optimizing-restriction-endonuclease-reactions • https://nebcloner.neb.com/#!/redigest
1c	• https://www.neb.sg/products/t1020-monarch-dna-gel-extraction-kit#Protocols,%20Manuals%20&%20Usage
3a	• https://www.thermofisher.com/document-connect/document-connect.html?url=https%3A%2F%2Fassets.thermofisher.com%2FTFS-Assets%2FLSG%2Fmanuals%2FMAN0012709_Rapid_DNA_Ligation_UG.pdf&title=VXNlciBHdWlkZTogUmFwaWQgRE5BIExpZ2F0aW9uIEtpdA
4b	• https://www.chem-agilent.com/pdf/strata/200314.pdf
6a	• https://www.qiagen.com/us/resources/resourcedetail?id=22df6325-9579-4aa0-819c-788f73d81a09&lang=en

### Transfection to generate lentiviral particles

**Timing: 4 days**

#### HEK293T cells are transfected to generate lentiviral particles.

***Note:*** The culture medium for HEK293T cells is DMEM supplemented with 10% fetal bovine serum and 1% penicillin-streptomycin.

***Note:*** The lentiviral particles are generated, using transfer plasmids and the pMD2.G, pRSV-Rev and pMDLg/pRRE packaging system. This is a 3rd generation, 4-plasmid system.

**Table:** 

Overview		
Day 1	Step 7	• Seeding of cells
Day 2	Step 8	• Transfection • Medium change at the end of the day
Day 4	Step 9	• Harvest lentiviral particles

7. Day 1: Seed 0.5 × 10^6^ HEK293T cells in 2 mL culture media into each well in 6 well plate.

***Note:*** The cells should be 70–80% confluent the next day (before transfection).

***Note:*** The cell number and transfection protocol below can be scaled by a factor of 0.4 if 12 well plate is used or by a factor of 2.5 if a 6 cm dish is used.

8. Day 2: Transfection
  a. Remove culture media.
  b. Wash cells with 1 mL PBS and add 1.92 mL OptiMEM media.
  c. Prepare the following mixes separately.
    i. Transfer vector mix:

**Table:** 

Reagent	Amount
Transfer vector	0.5 μg
pMDLg/pRRE vector	0.24 μg
pRSV-Rev vector	0.12 μg
pMD2.G vector	0.14 μg
OptiMEM media	Complete to 40 μL
    ii. Endofectin Lenti mix

**Table:** 

Reagent	Amount
Endofectin Lenti (GeneCopoeia Cat# EF001)	3 μL
OptiMEM media	Complete to 40 μL
  d. Add the Endofectin Lenti Mix to the transfer vector mix dropwise while vortexing. Leave the mixture at 20°C–22°C for 15 min.
    **CRITICAL:** Avoid vortexing the mixture after the incubation as it might disrupt the complexes.
  e. Add the mixture dropwise to the cells in 6-well plate.
    **CRITICAL:** Avoid adding all of the mixture to the cells at one spot. It might affect the cell viability.
  f. At the end of the day (∼7–8 h), aspirate out the infection medium and add 1 mL fresh OptiMEM to the cells. Continue incubation at 37°C for 48 h.

***Note:*** The medium can also be changed the next day (∼24 h later). Depending on the transfection reagent, the timing for the media change might be different. It is advised to check the manual of the transfection reagent chosen.

9. Day 4: Harvest lentiviral particles.
  a. Harvest the media. Spin down at 300 × *g* for 5 min to pellet down cell debris. Aliquot the supernatant containing the lentiviral particles into tubes.
    ***Note:*** The viral particles can be harvested using 0.45 μm filters. Do not use 0.22 μm filters as it will remove the viral particles.
  b. Store the tubes at −80°C.

### Transduction to generate S protein- expressing cells

**Timing: 4 days**

#### HEK293T cells are transduced to generate cells expressing the full length S protein.

***Note:*** The transduction protocol described has been optimized using HEK293T cells. It has also been similarly applied to HEK293, EL4 and K562 cells. However, do ensure that all samples, that are going to be compared, are analyzed using the same cell line, as different cell lines might have different glycosylation modifications of the spike protein, affecting the antibody binding.

**Table:** 

Overview		
Day 1	Step 10	• Seeding of cells
Day 2	Step 11	• Transduction • Medium change at the end of the day
Day 4	Step 12	• Sort for GFP-positive cells

10. Day 1: Seed 0.12 × 10^6^ HEK293T cells into each well in 12 well plate.

***Note:*** The cells should be 70–80% confluent the next day (before transduction).

11. Day 2: Transduction
  a. Add polybrene to viral supernatant (final concentration in well 8 μg/mL).
    ***Note:*** Always include a negative control (a well where fresh culture media is added in place of the viral supernatant).
    ***Note:*** It is recommended to determine the viral titer by qRT-PCR or p24 ELISA before transduction as different production lots might have different yields of virions. We have found that, if the transgene is with a phenotype detectable by flow cytometry (such as eGFP in this case), it is a better method of quantifying the viral titer than p24 ELISA or qRT-PCR (both of which measure incomplete/non-functional virus components in addition to functional virions). If it is the first time performing transduction, it is advisable to perform a few conditions by varying the amount of viral supernatant per well: eg. 2 μL, 20 μL, 200 μL.
  b. Add the polybrene/viral supernatant mixture to the well.
  c. Spin at 1200 × *g* for 1 h at 32°C.
    ***Note:*** Pre-warm the centrifuge to 32°C before use.
  d. Incubate at 37°C.
  e. At the end of the day (∼7–8 h), aspirate out the infection medium and add fresh culture media to the cells. Continue incubation at 37°C for 48 h.

***Note:*** If the cells are near 100% confluency, passage the cells accordingly.

12. Day 4: Sorting for transduced cell expressing full length S protein.
  a. Harvest the cells by re-suspending PBS.
    ***Note:*** Avoid using trypsin to detach the cells. PBS or 2mM EDTA can be used to detach the cells.
  b. Sort for eGFP-positive cells.
  c. Expand and cryopreserve till use.

***Note:*** The expression of the spike protein can be validated using a commercial monoclonal antibody against the spike protein, eg. Thermo Fisher Scientific #703958.

## Key resources table

**Table:** 

REAGENT or RESOURCE	SOURCE	IDENTIFIER
**Antibodies**		

Anti-human IgG Alexa Fluor 647 (used at 1:600 dilution)	Thermo Fisher Scientific	Cat# A21445; RRID:AB_2535862
Anti-human IgM Alexa Fluor 647 (used at 1:600 dilution)	Thermo Fisher Scientific	Cat# A21249; RRID:AB_2535817
Anti-human IgA Alexa Fluor 647 (used at 1:600 dilution)	BioLegend	Cat# 411502; RRID:AB_2650697
Anti-mouse IgG Alexa Fluor 647 (used at 1:600 dilution)	Thermo Fisher Scientific	Cat# A21235; RRID:AB_2535804
Anti-human IgG1 (used at 1:600 dilution)	Thermo Fisher Scientific	Cat# MA1-34581; RRID:AB_11004658
Anti-human IgG2 (used at 1:600 dilution)	BioLegend	Cat# 411102; RRID:AB_2686940
Anti-human IgG3 (used at 1:600 dilution)	BioLegend	Cat# 411302; RRID:AB_2686942
Anti-human IgG4 (used at 1:600 dilution)	Thermo Fisher Scientific	Cat# A10651; RRID:AB_2534053
Anti-spike monoclonal antibody (used at 1:1000 dilution)	Thermo Fisher Scientific	Cat# 703958; RRID:AB_2866477

**Bacterial and virus strains**		

XL10 gold ultracompetent bacterial cells	Agilent	Cat# 200314
XL10 bacterial cells harboring pHIV-SARS-CoV-2-SP-eGPF	Generated in this study	NA

**Biological samples**		

Plasma samples from symptomatic COVID-19 patients	N/A	IRB# 2020/00091
Plasma samples from healthy donors	N/A	IRB# 2017/2806 and IRB# 04-140

**Chemicals, peptides, and recombinant proteins**		

XbaI	NEB	Cat# R0145S
BamHI	NEB	Cat# R0136S
1 Kb DNA ladder	NEB	Cat# N3232S
Monarch® DNA Gel Extraction Kit	NEB	Cat# T1020S
Rapid ligation kit	Thermo Fisher Scientific	Cat# K1422
Phusion DNA polymerase	Thermo Fisher Scientific	Cat# F530L
dNTP	Thermo Fisher Scientific	Cat# R0481
Agarose	1st BASE	Cat# BIO-1000-500g
LB agar	1st BASE	Cat# CUS-4003-400mL
LB broth	Gibco	Cat# 10855-021
Ampicillin	Sigma-Aldrich	Cat# A0166
QIAprep Spin Miniprep kit	QIAGEN	Cat# 27104
Dulbecco's Modified Eagle Medium (DMEM)	HyClone	Cat# SH30022.01
Fetal Bovine Serum (FBS)	HyClone	Cat# SV30160.03HI
Penicillin-Streptomycin	Gibco	Cat# 15140-122
OptiMEM media	Thermo Fisher Scientific	Cat# 31985070
EndoFectin Lenti	GeneCopoeia	Cat# EF001
Polybrene	Sigma-Aldrich	Cat# H9268
Propidium iodide	Sigma-Aldrich	Cat# P4170
EDTA	1st BASE	Cat# BUF-1052-500mL-pH8.0
TAE	1st BASE	Cat# BUF-3000-50X1L
PBS	Gibco	Cat# 20012027

**Experimental models: cell lines**		

HEK293T	ATCC	Cat# CRL-3216
HEK293T expressing full-length S protein	Generated in this study	NA

**Oligonucleotides**		

EF1aFor (Table 1)	Integrated DNA Technologies	EF1aFor
SPseqF1 (Table 1)	Integrated DNA Technologies	SPseqF1
SPseqF2 (Table 1)	Integrated DNA Technologies	SPseqF2
SPseqF3 (Table 1)	Integrated DNA Technologies	SPseqF3
SPseqF4 (Table 1)	Integrated DNA Technologies	SPseqF4
SPseqR1 (Table 1)	Integrated DNA Technologies	SPseqR1
IRESrev (Table 1)	Integrated DNA Technologies	IRESrev

**Recombinant** DNA		

pHIV-eGFP	Addgene	Cat# 21373
pMD2.G	Addgene	Cat# 12259
pMDLg/pRRE	Addgene	Cat# 12251
pRSV-Rev	Addgene	Cat# 12253
pHIV-SARS-CoV-2-SP-eGPF	Generated in this study	NA

**Software and algorithms**		

FlowJo	Tree Star	NA
pROC library	R version 3.6.4	NA

**Others**		

6-Well plates	Thermo Fisher Scientific	Cat# 140675
12-Well plates	Thermo Fisher Scientific	Cat# 150628
96 V-bottomed well plates	Thermo Fisher Scientific	Cat# 249570
LSR II 4 laser	BD Biosciences	NA
Nanophotometer	IMPLEN	Cat# N60

## Step-by-step method details

### Flow cytometry-based assay to detect antibodies specific for SARS-CoV-2 S protein

**Timing: 2 h**

All patients’ plasma/serum samples are diluted 1 in 100, while all secondary and tertiary antibodies are diluted 1 in 600.

1. Harvest HEK293T cells expressing S protein.
  a. Remove spent media.
  b. Wash with PBS.
  c. Detach with ice-cold 2 mM EDTA for 1–2 min.
  d. Wash twice with PBS by centrifugation at 300 × *g* for 5 min.

**CRITICAL:** Avoid using trypsin to detach the cells. The S protein is sensitive to trypsin cleavage. Similarly, avoid using cell scrapper, as it might affect the expression of the S protein on the cell surface.

2. Seed 0.15 × 10^6^ cells into each well in 96 V-bottomed well plates.
  a. All samples are analyzed in technical duplicates.
  b. Pellet the cells down by centrifugation at 300 × *g* for 5 min.

***Note:*** The assay has been optimized for 0.1–0.25 × 10^6^ cells/well. However, due to cell loss (through centrifugation), we recommend at least 0.15 × 10^6^ cells/well.

3. Re-suspend cells in diluted plasma/serum samples.
  a. Dilute the samples at 1:100 in FACS buffer (10% FBS diluted in PBS) prior to addition to cells.
  b. Ensure that negative and positive control samples are also included. Eg. Anti-spike monoclonal antibody (e.g., Thermo Fisher Scientific #703958) can be used as positive controls and healthy control plasma/sera can be used as negative controls.
4. Incubate at 4°C for 30 min in the dark.
5. Wash twice with PBS by centrifugation at 300 × *g* for 5 min.
6. Re-suspend cells in diluted secondary antibody incubation.
  a. Dilute the secondary antibodies at 1:600 in FACS buffer prior to addition to cells.
  b. For IgG, IgM and IgA detection, the secondary antibody is anti-human IgG, anti-human IgM and anti-human IgA Alexa Fluor 647 antibodies in FACS buffer with 1 μg/mL propidium iodide.
  c. For IgG subclasses detection, the secondary antibody is mouse anti-human IgG1, IgG2, IgG3 and IgG4 antibodies in FACS buffer.

***Note:*** Other fluorophores, other than Alexa Fluor 647, can also be used. One other possible option is Alexa Fluor 405, which have little compensation issues with the GFP-positive cells and the propidium iodide staining. We have chosen Alexa Fluor 647 as there is also little compensation issues with the GFP-positive cells and the propidium iodide staining.

***Note:*** In place of propidium iodide, DAPI can also be used for staining to differentiate live/dead cells. Alternatively, other live/dead viability dyes may be used.

**Figure 2:**
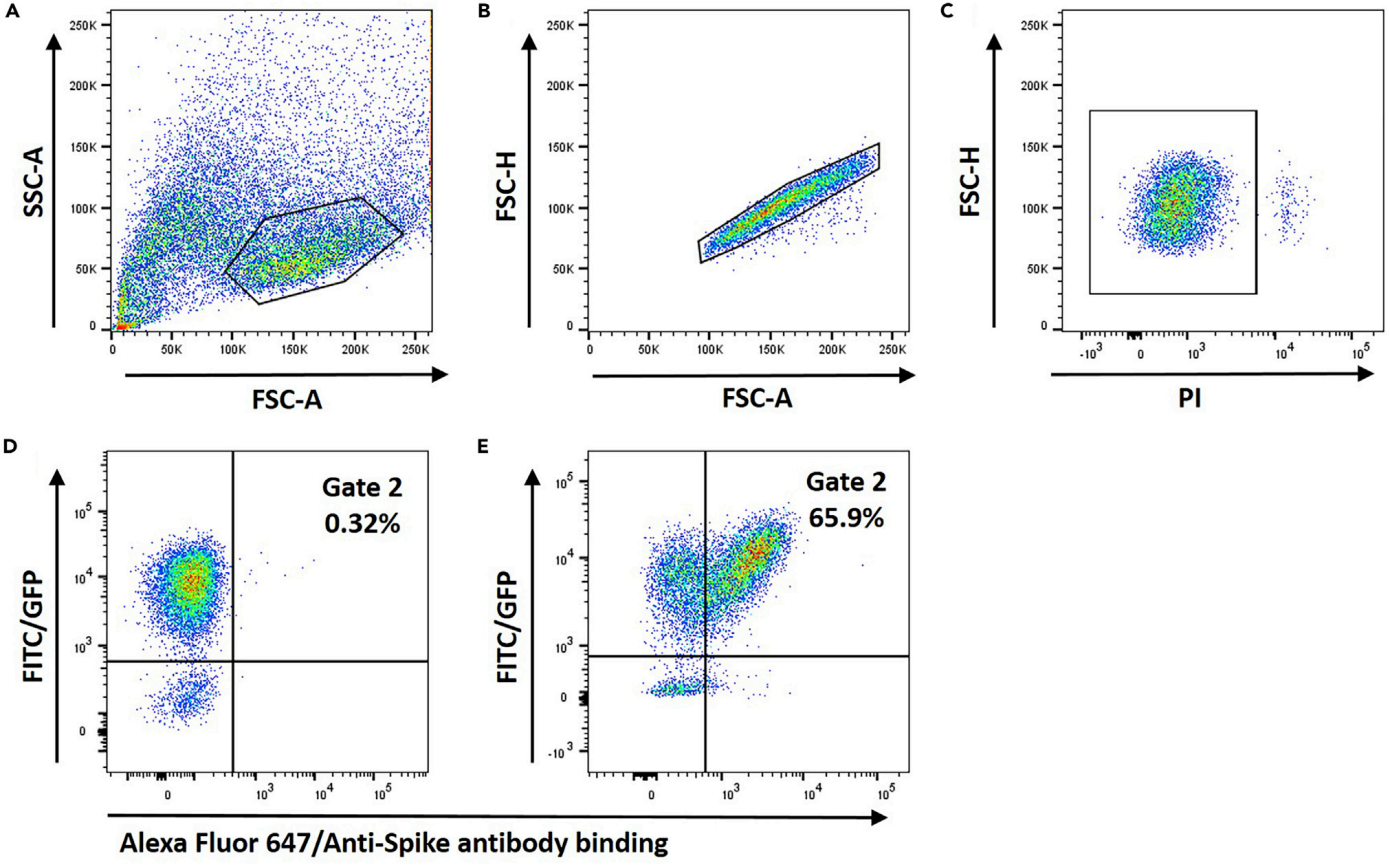
FACS plot analysis Cells were gated on: (A) FSC-A/SSC-A to exclude cell debris, (B) FSC-A/FSC-H to select for single cells, (C) FSC-H/PI to select for live cells (PI-negative population), (D, E) FITC/Alexa Fluor 647 for specific antibody binding. Binding is determined by the percentage of GFP-positive S protein-expressing cells that are bound by specific antibody, indicated by the events that are Alexa Fluor 647- and FITC-positive (Gate 2). (D) PBS control; (E) COVID-19 patient plasma, 1:100 diluted.

***Note:*** Cells are read on LSR4 laser (BD Biosciences), however, the cells can be read on any other cytometers with the following specifications (Table 3).

7. Incubate at 4°C for 30 min in the dark.
8. Wash twice with PBS by centrifugation at 300 × *g* for 5 min.
9. For IgG and IgM detection, add 100 μL FACS buffer to the well. Re-suspend well and analyze by flow cytometry.
10. For IgG subclasses detection, re-suspend cells in diluted tertiary antibody incubation.
  a. Dilute the secondary antibodies at 1:600 in FACS buffer prior to addition to cells.
  b. The tertiary antibody is anti-mouse Alexa Fluor 647 antibodies in FACS buffer with 1 μg/mL propidium iodide (PI; Sigma-Aldrich #P4170).
11. Incubate at 4°C for 30 min in the dark.
12. Wash twice with PBS by centrifugation at 300 × *g* for 5 min.
13. Add 100 μL FACS buffer to the well. Re-suspend well and analyze by flow cytometry.
  a. Cells were gated on: (1) FSC-A/SSC-A to exclude cell debris (Figure 2A), (2) FSC-A/FSC-H to select for single cells (Figure 2B), (3) FSC-A/PI to select for live cells (PI-negative population, Figure 2C), (4) FITC/Alexa Fluor 647 (Figures 2D–2H). Binding is determined by the percentage of GFP-positive S protein-expressing cells that are bound by specific antibody, indicated by the events that are Alexa Fluor 647- and FITC-positive (Gate 2). A sample is defined as positive when the binding is more than mean + 3SD of the healthy controls.

**Table 3:** Cytometer specifications

Laser (wavelength)	Fluorochrome (marker)	BP filter	LP filter	Detection range	PMT voltage used
Blue (488 nm)	SSC	488/10			345
Blue (488 nm)	FSC				273
Blue (488 nm)	PE-Texas red (PI)	610/20	595LP	600–620 nm	535
Blue (488 nm)	FITC (GFP)	530/30	505LP	515–545 nm	480
Red (633 nm)	Alexa Fluor 647 (anti-spike antibody staining)	660/20		650–670 nm	550

## Expected outcomes

Using this assay, we are able to analyze the S protein-specific antibody profile of symptomatic and asymptomatic COVID-19 patients (Goh et al., 2021). While the antibody levels are lower in asymptomatic patients, the assay is highly sensitive and detects 97% of the asymptomatic infections. We also found that IgG1 is the dominant IgG subclass in both symptomatic and asymptomatic patients.

## Quantification and statistical analysis

### Quantification of S protein antibody by flow cytometry

Specific antibody binding to cells was determined by LSRII 4 laser (BD Biosciences) and analyzed using FlowJo (Tree Star).

1. Gate the cells based on the following:
  a. Forward (FSC) and side (SSC) scatter parameters, FSC-A/SSC-A, to exclude cell debris (Figure 2A)
  b. FSC-A/FSC-H, to select for single cells (Figure 2B),
  c. FSC-A/PI, to select for live cells (PI-negative population, Figure 2C),
    ***Note:*** We use PI dye to stain for dead cells.
  d. FITC/Alexa Fluor 647, to determine the level of specific S protein binding (Figures 2D–2H). Binding is determined by the percentage of GFP-positive S protein-expressing cells that are bound by specific antibody, indicated by the events that are Alexa Fluor 647- and FITC-positive (Gate 2).

2. Define a sample as positive when the binding is more than mean + 3SD of the healthy control individuals. The thresholds using the healthy control readings is based on the normal-like distribution of the healthy control reading where a mean + 3SD threshold would mean that there is less than a 0.13% chance of a false positive.

***Note:*** In Goh et al. (Goh et al., 2021), our sample size of healthy control individuals was 22 and the Receiver Operating Characteristic (ROC) curves were constructed from each of the antibody binding with the healthy control individuals and SARS-CoV-2 patients as the true negatives and true positives respectively using the pROC library in R version 3.6.4.

## Limitations

Similar to all serological assays, the risk of false positive diagnosis is one of the limitations of the assay. However, the assay consists of seven tests (IgM, IgA, IgG, and four IgG subclasses), allowing internal validation. Nevertheless, borderline positive results should be interpreted with caution. One other limitation of the SFB assay is the need for advanced planning. The assay is a cell-based assay, hence the dependence on cell culture requires careful planning ahead to ensure sufficient cell count. This limits the application of the assay for HTS. We suggest performing different serological assays in parallel: (1) this would complement each other to provide better diagnosis, and (2) other serological assays that allows high throughput screening application, could serve as the first round of screening, and the more sensitive SFB assay could provide confirmation and further investigation of borderline/discrepant samples. As the SFB assay is a cell-based FACS assay, the acquisition of the samples can time-costly, especially when the sample size is large.

## Troubleshooting

### Problem 1

Inefficient digest of vector backbone (step 1 of before you begin).

### Potential solution

Set up the digest reaction with 10 U of enzymes in excess per 5 μg vector.

### Problem 2

No colonies following DNA ligation (step 3 and 4 of before you begin).

### Potential solution

The DNA ligation can be optimized by:

Incubating the ligation reaction at 16°C for 12–16 h.
Ensuring efficient digest of the vector backbone and insert.

### Problem 3

Low viral titer (step 9 of before you begin).

### Potential solution

Concentrate using a Vivaspin-20 centrifugal device (100 kD MWCO, Sartorius # VS2042).

### Problem 4

Insufficient cells for acquisition on the cytometer (step 13 of step-by-step method details).

### Potential solution

Possibly due to significant cell loss throughout the assay. In this case, increase the cell number per well to 0.25 × 10^6^ cell per well.

### Problem 5

No binding, as indicated by absence of Alexa Fluor 647 staining (step 13 of step-by-step method details).

### Potential solution

Possibly because the secondary or tertiary antibodies was left out. In this case, re-stain with secondary antibody incubation. Ensure that positive control samples are included.

## Resource availability

### Lead contact

Further information and requests for resources and reagents should be directed to and will be fulfilled by the lead contact : Laurent Renia, Infectious Diseases Laboratories (ID Labs), A∗STAR, 8A Biomedical Grove, #03-15, Immunos Building, Biopolis, Singapore 138648; Tel: +65 64070005; Fax: +65 6464 2056; Email: renia_laurent@idlabs.a-star.edu.sg.

### Materials availability

All unique/stable reagents generated in this study are available from the lead contact with a completed Materials Transfer Agreement.

### Data and code availability

This study did not generate any datasets/code.

## References

[bib1] Goh Y.S., Chavatte J.M., Lim Jieling A., Lee B., Hor P.X., Amrun S.N., Lee C.Y., Chee R.S., Wang B., Lee C.Y. (2021). Sensitive detection of total anti-Spike antibodies and isotype switching in asymptomatic and symptomatic individuals with COVID-19. Cell Rep. Med..

